# Panic, denial, and the search for “magic remedies:” the various social responses to covid-19 in Peru

**DOI:** 10.1590/S0104-59702023000100031

**Published:** 2023-08-14

**Authors:** Jorge Lossio

**Affiliations:** i Senior Professor, Department of Humanities/Pontificia Universidad Católica del Perú. Lima – Peru jorge.lossio@pucp.pe

**Keywords:** Covid-19, Peru, History, Social responses, Covid-19, Perú, Historia, Respuestas sociales

## Abstract

Between March 2020 and March 2022, more than 213,000 Peruvians died of covid-19. In this research note, we will identify and analyze the various social responses to covid-19 in Peru: denial, panic, search for culprits, search for “magic remedies,” and, in some sectors, mistrust towards the State and science. We argue that these social responses have been common throughout history, both in pandemics prior to the development of the bacteriological era and in the most recent ones. We intend to show that, in Peru, scientific ideas around health and disease coexist with religious ideas, conspiracy theories and traditional knowledge.

## Resumen

Entre marzo de 2020 y marzo de 2022, más de 213.000 peruanos fallecieron por covid-19. En esta nota de investigación, identificaremos las diversas respuestas sociales a la covid-19 en Perú: negación, pánico, búsqueda de culpables, búsqueda de “remedios mágicos” y, en algunos sectores, escepticismo y desconfianza hacia el Estado y la ciencia. Argumentamos que estas respuestas sociales han sido comunes a lo largo de la historia, tanto en las pandemias anteriores al desarrollo de la era bacteriológica como en las más recientes. Pretendemos mostrar que en el Perú conviven ideas científicas en torno a la salud y la enfermedad con ideas religiosas, teorías conspirativas y saberes tradicionales.

Between March 2020 and March 2022, more than 213,000 Peruvians died of covid-19. Peru was one of the countries with the highest mortality rate from the virus in the world. In this article, we will identify the various social responses to covid-19 in Peru: lethargy, panic, search for culprits, the appearance of charlatans and “magic remedies,” and, in some sectors, denial, skepticism, and mistrust of the State and science.

## The initial indifference

Between December 2019 and early March 2020, the Peruvian society reacted mostly with indifference to the news of the novel virus circulating in China. Life continued, unchanged: international travel, the influx to beaches, soccer stadiums, bars and clubs remained as usual. For instance, at the beginning of March 2020, a soccer match was held between the most traditional teams of Lima, Universitario de Deportes and Alianza Lima, in the Monumental Stadium, which was attended by more than 50 thousand spectators. This occurred even though, since the end of December 2019, the national and international press reported about a virus that had emerged from China’s Wuhan market and was spreading rapidly. Despite living in a globalized world, the initial attitude of indifference was based on the idea that the virus would not reach Peru. This was because either it was a distant and foreign problem, or the press was exaggerating the magnitude of the possible health crisis (the widespread idea in the country of being faced with “smokescreens” produced to divert the attention from other problems).

As for international health organizations, initially, the message about the severity of the crisis was not clear either. It was not until January 30, 2020, that the World Health Organization (WHO) declared that there was a health emergency of international concern. In the case of the Peruvian State, the first action plan in response to the coronavirus was presented on January 31, 2020, the main purpose of which was to prevent the virus from reaching Peru. At the time of the pandemic outbreak, the minister of Health was María Elizabeth Jacqueline Hinostroza Pereyra, a surgeon graduated from Universidad de San Marcos and General of the Peruvian National Police, and the president of the Republic was Martín Vizcarra. In February 2020, the temperature of passengers arriving at Jorge Chavez International Airport began to be taken and information campaigns on the novel coronavirus were organized by the State for those who were going to travel internationally. However, the increase of cases in China and the occurrence of the virus in other Asian countries, Europe and the United States began to generate greater concern in society and more restrictive measures by the State.

## Responses to quarantine: panic, passivity, and resistance

On March 6, 2020, the then president of the Republic, Martín Vizcarra, confirmed the first covid-19 case in the country. On March 10, 2020, President Martín Vizcarra announced a 14-day home quarantine for all travelers coming from Spain, France, Italy, and China, the countries with the highest number of cases at the time. On March 11, 2020, the government announced the closure of public and private schools. That same day, WHO declared that the world was facing a pandemic. On March 12, 2020, the Peruvian government decreed a national health emergency for a period of 90 days (this period was extended for two more years). Health Minister, Elizabeth Hinostroza, emphasized the importance of social isolation and the government banned public shows with more than 300 people. On March 14, the Ministry of Culture of Peru suspended activities in every museum, library, and theater. The first death in the country was confirmed on March 19, 2020, in Lima. In the second week of March, instances of panic began. For example, agglomerations in markets, supermarkets, and shopping centers were caused by people exaggeratedly buying products such as toilet paper, personal hygene products, bottles of water, and non-perishable food, such as rice, noodles, and sugar. Some supermarkets in Lima began to place limits on the sale of these products (Coronavirus..., 14 Mar. 2020).

On Sunday, March 15, 2020, President Vizcarra announced a strict national quarantine for two weeks (although it finally lasted more than a hundred days). During this quarantine, people could only leave their homes for essential shopping (pharmacies, markets) and banking. A curfew was also ordered nationwide (in Lima, it was from 20h to 5h during most of the quarantine; other regions had some more curfew hours). An order of absolute immobility was given for Sundays. An unprecedented experience in Peruvian history. A large sector of the population perceived this as a drastic but necessary measure (Ipsos, abr. 2020), and resignedly accepted this dramatic change in daily life. Houses became homes, schools and workplaces, all at the same time. This measure “ended” the in-person social life of Peruvians, in some cases replaced by virtual meetings (birthdays, weddings, parties, over Zoom). On March 20, 2020, the then minister of Health, Elizabeth Hinostroza, was removed from office. Víctor Zamora Mesía, epidemiologist and professor at Universidad Peruana Cayetano Heredia, was appointed in her place. President Martín Vizcarra explained this change stating he needed a minister specialized in public health to fight the pandemic ( Asensio, 2020 ).

The initial support for this quarantine was declining over time for various reasons, and by June 2020, hundreds of thousands of people had already been seized by the police for disobeying State-imposed isolation measures. First, because of the duration of the measure. In the beginning, it was announced that it would last 15 days, but it ended up being more than one hundred days of strict quarantine. There was no clear timetable for the end of the quarantine, but it was announced that it would be extended every 15 days. At some point, it seemed endless. Secondly, the economic factor. Only a small percentage of Peruvians could work from home on the online modality and continue to receive their salary. A high percentage of the population makes a living from daily street vending and cannot afford to stay at home. Thirdly, there was a sector of the population that disobeyed the quarantine by throwing clandestine parties or playing clandestine soccer matches. This occurred in all social strata and nationwide. In this case, it is assumed that those people were skeptical of the news about the virus or had a fatalistic approach to death (Coronavirus..., 7 Sep. 2020).

As for the search for culprits in the health crisis, it could be said that two were found. First, Chinese eating habits were blamed for being the root cause of the disease. This is linked to the fact that the pandemic emerged from the Wuhan seafood market and to a series of long-standing prejudices in the country towards the health, hygiene and eating habits of the Chinese population. The press initially disseminated the idea that covid-19 had emerged from the consumption of pangolin or bats in China. Throughout Peruvian history, it has not been unusual to blame the Chinese for the outbreak of epidemics or pandemics: this was the case with yellow fever in the nineteenth century and with the bubonic plague in the early twentieth century. As to why the quarantine did not work as expected (despite the strict measures taken by the State, cases continued to rise), those who disobeyed quarantine and isolation (street vendors or those who threw clandestine parties) were blamed. At this point, it should be noted that various health specialists (historians, sociologists, health officials, epidemiologists) rejected anti-Chinese racism in various media and questioned the blaming of street vendors as responsible for the failure of State measures.

In the need to look for blame for the health crisis within the less well-off groups, we see a repetition of patterns from the past. The same thing happened with the yellow fever epidemics in the nineteenth century and the Chinese, or with aids and homosexual men in the 1980s.

On June 30, 2020, President Martín Vizcarra announced the end of the national quarantine to give way to a strategy of targeted quarantine. The obligation to remain at home would be maintained throughout the country for children under 14 years of age, people over 65 years of age and people with comorbidities. The end of the general quarantine included the resumption of land and air transportation across the country, and the resumption of in-person service with limited capacity in shops, restaurants, and other establishments.

## The search for magic remedies

It has been usual throughout history that, in times of epidemics and pandemics, people resort to charlatans, religious images, or home remedies. That is, if we review past experiences like the yellow fever epidemics in the nineteenth century, or the bubonic plague in the early twentieth century, people did not listen only to university physicians. The health market was broad and diverse. Western science was not the only way to explain or understand health and disease. However, covid-19 has shown that this diverse health market still exists in the twenty-first century, and that the opinions of health officials and scientists are not the only ones considered as valid. In fact, many times, the Peruvian population ignored the recommendations of health officials and chose to take medication based on information circulating on social media or based on personal beliefs with no scientific basis (Salud..., 29 Apr. 2021). For example, the use of ivermectin was popular in Peru; hydroxychloroquine, azithromycin, chlorine dioxide, and *cañazo* were also used; and pseudoscientists promised miraculous cures through concoctions and exorcisms. This shows us how, despite the important discoveries made during the bacteriological era of the nineteenth century, at a popular level there are multiple ideas that coexist around health and disease.

In the case of ivermectin, it must be said that the initial messages from health officials and physicians were unclear or contradictory. There were physicians who supported the use of ivermectin to prevent covid-19, and it was even used in some State campaigns against this disease (Coronavirus..., 27 June 2021). The consumption of this deworming drug was so high that, according to newspaper *El Comercio* , “during 2020, Peru imported 500% more ivermectin than the amount reported the previous year” (Coronavirus..., 15 Feb. 2021). As Professor Marcel Velázquez tells us, when the quarantine was in full swing in Nauta, evangelical missions massively gave ivermectin shots. According to his account, in some evangelical groups, the arrival of the pandemic was seen as a confirmation of biblical prophecies (Velázquez, 2020, p.89).

However, even when the international medical community and the Peruvian State started to communicate more clearly their position against the use of ivermectin, since the beginning of 2021, a part of the Peruvian population continued to use this resource as a preventive measure against covid-19. An idea that began to circulate on social media and became popular was that there was a plot by drug companies against the use of ivermectin because of its low price, which prevented them from profiting from the disease. This phenomenon did not occur in Peru only, but globally, and Facebook groups appeared in several countries, promoting the consumption of this anti-parasitic drug (Grupos…, 28 Sep. 2021).

Another “remedy” used was chlorine dioxide. Unlike ivermectin, in this case, the medical community and health officials were clear from the beginning in their rejection. There were some misleading actions, such as the decision of the Peruvian Congress to form a commission to investigate the effects of chlorine dioxide (Congreso…, 13 May 2021) and some isolated voices of physicians who were in favor of its use, although they were a minority. The Ministry of Health of Peru asked not to use chlorine dioxide against covid-19 and warned of the dangers of its use: “Consumption of chlorine dioxide or sodium chlorite can seriously harm people’s health because it is not a drug but a powerful bleaching agent. Therefore, it does not cure any disease” (Perú, 23 July 2020). In July 2020, the then Minister of Health, Pilar Mazzetti, stated that its use was not approved and that there was no clinical trial or scientific evidence in favor of its use against covid-19 (Ministerio de Salud..., 23 July 2020). However, there were people who used it, which shows the limits of the recommendations of health officials and how society gets information through other media about health and disease.

There were also cases of charlatans. For instance, the press reported the case of Edelfi Bereche, a Lima-based “healer” who charged 250 soles and promised to cure his “patients” from covid-19; therefore, he was arrested on July 12, 2020. That same month, “Doctor Teo,” a healer from Trujillo who claimed to be sent by Godand to be able to cure from covid-19, was imprisoned for selling his patients a concoction that could cost up to 400 soles. Some religious leaders also took advantage of people’s fear and offered miraculous cures for the coronavirus, as was the case of an evangelical pastor in Lambayeque, who claimed covid-19 was caused by the demon and offered exorcisms to “cure” his followers. The pastor held events in which many people without masks gathered in an enclosed space. Just as in the case of the smallpox epidemics of the eighteenth century, a sector of the population turned to religion, seeking to mitigate the impact of the disease.

Throughout history, processions and prayer chains have been common in times of epidemics and pandemics. As happened in the past, in the case of the covid-19 pandemic, religious leaders and civil society organized days of fasting and prayer to fight the disease. As stated by Marcel Velázquez (2020 , p.87) in his book *Hijos de la peste* , in the middle of the quarantine, to ask for protection against the virus, human chains of prayer were formed in Iquitos, masses were celebrated in Lima hospitals, the figure of Christ was carried through the city of Huancavelica, and the Virgin of Candelaria was taken out around Puno. In fact, the Virgin Mary has been considered a protective figure against epidemics since colonial times. Another issue to consider is the cultural diversity in Peru. That is, there are multiple world-views and traditions regarding health (coastal, Andean, Amazonian) and, therefore, multiple ways of explaining the origin of diseases and causes of illnesses that do not necessarily follow scientific guidelines. The use of ginger, eucalyptus or matico was recommended at the popular level to cure covid-19.

This shows how, despite the advance of science and the greater presence of the State in health and education compared to previous centuries, the acts and opinions valued by society on health and disease issues include a wide range of actors and are not guided solely by the State or scientific recommendations ( Velázquez, 2020 , p.155). What opinions are considered valid on health issues? As has happened throughout history, opinions vary widely, including those of religious leaders, charlatans, pseudo-scientists, healers, among others. Moreover – and this deserves specialized work – the anti-science discourse may have been strengthened in the twenty-first century by social media, where extravagant or negationist ideas find enough echo to influence a broad section of the population.

On the other hand, there were valuable efforts to combine traditional medical knowledge with Western medicine. In Ucayali, for instance, a group of young people formed the Matico Squad, which focused on providing care to the indigenous populations of the Amazon, either using plants such as matico or by seeking greater presence of the State and Western medical assistance (Ucayali…, 16 May 2020). The use of matico and other traditional medicinal plants during the pandemic was common in the Amazonian region. Although there is no doubt that there are plants with healing properties for certain illnesses that are known and used since ancient times in the Andes and in the Peruvian Amazon, there are no studies that can certify that plants such as matico or eucalyptus are useful against covid-19. The use of these plants during the pandemic shows the permanence of ancient medical traditions and the absence of a State capable of offering scientifically validated medical treatments.

## Social reactions to the covid-19 vaccine

The covid-19 pandemic has shown that, although anti-scientific postures still exist and part of the population does not trust the State, health officials, and scientists, the vaccine is a public health tool with widespread popular acceptance in the country. It should also be noted that there was initially some reluctance toward vaccines made in China, partly because of scandals such as “vacunagate” and the fake news that circulated in the media and social media.

The vaccination process against covid-19 in Peru had a turbulent and hopeless start. By mid-2020, President Martín Vizcarra created the “Vaccine Squad”, which contacted several laboratories, the clinical studies of which were in their final phase, and issued emergency decree n.110-2020 dated August 6, 2020, establishing “extraordinary measures to facilitate and ensure the acquisition, conservation and distribution of vaccines against covid-19.” This decree would allow the State to make contracts with private companies and international organizations, as well as to make advance payments for the purchase of vaccines. At the same time, phase-III clinical trials were promoted in the country. Collaboration with vaccine company trials were to function as a way of facilitating subsequent access to their vaccines, since relationships and contacts were already established. This was the case of the Chinese laboratory Sinopharm, which worked in collaboration with Universidad Peruana Cayetano Heredia.

However, in the second half of 2020, the purchase of vaccines did not materialize, which began to generate indignation, despair, and much criticism of the functioning of the State. Hundreds of thousands of Peruvians traveled to the United States to get vaccinated, and libertarian and right-wing leaders in Peru claimed that the private sector should be left to lead the vaccination campaign. The ideological bias did not allow us to recognize that one of the most important advances in health had been the increased presence of the State in health and the creation of institutions such as the national vaccine institute or the expanded program of immunizations; also, that the vaccination campaigns pushed by the Peruvian State had led to the end of diseases such as smallpox and polio in the country.

Amidst this health crisis, in November 2020, President Martín Vizcarra was removed from office by the Congress of the Republic. President Vizcarra’s vacancy caused rejection from a sector of the population, which took to the streets in several cities of the country to protest against the new president, Manuel Merino, who had assumed the presidency since he was the president of the Congress of the Republic. Manuel Merino appointed Abel Salinas Rivas, who was in office for a few days, as minister of Health. Finally, due to the magnitude of the protests, Manuel Merino resigned from the presidency on November 15, 2020, and the Congress appointed engineer and academic Francisco Sagasti to the position of president of the Republic. Francisco Sagasti emphasized two aspects: buying vaccines and organizing national elections.

In December 2020, President Sagasti announced that he had not found any purchase contracts for the supply of vaccines, but merely preliminary agreements. To make matters worse, a scandal broke out in February 2021, when it was reported on television and press that former President Martín Vizcarra had received Sinopharm’s vaccine candidate while clinical trials were still underway. Vizcarra, who was then running for Congress, responded that the vaccine he had received was given within the framework of the study, in which he had volunteered. Thus began the scandal that would be called “vacunagate” by the press (Coronavirus…, 18 Feb. 2021). Health Minister Pilar Mazzetti resigned on February 12, 2021, and was replaced by Oscar Ugarte. The “vacunagate” scandal has led many people to question how clinical trials were conducted, and thus the safety of vaccines. Mistrust was mainly towards China’s Sinopharm vaccine.

From that point on, there was a major shift in the vaccine purchase campaign. On January 11, 2021, supreme decree n.002-2021-SA was approved, creating the Regulations for the Conditional Sanitary Registry of Medicines and Biological Products, the legal framework needed for the purchase of vaccines. On January 6, 2021, Sagasti announced that an agreement had been signed with the AstraZeneca laboratory for the delivery of 14 million doses due from September, as well as one with Sinopharm for 38 million doses, of which a first batch of one million would arrive in Peru in February 2021. A preliminary agreement was signed with Johnson & Johnson on January 16, 2021, and a contract with Pfizer for 20 million doses was signed on February 4. The arrival of the first 300,000 Sinopharm vaccines on February 7, 2021, allowed the start of the vaccination campaign, the first vaccinated being first-line physicians. That same day, the president of the Republic was vaccinated too, as a way of showing that vaccines were safe. The images of the vaccines arriving in Peru generated national excitement.

As vaccine shipments became more frequent and the stock thereof increased, the government accelerated the pace of vaccination. Although the initial fears subsided, the State had to deal with the fake news broadcast via social media blaming vaccines for being sterilization tools, causing other diseases, implanting chips in us, and other fake information. A survey conducted by the public opinion research firm Ipsos revealed that only 57% of respondents were willing to be inoculated, compared to 39% who were opposed (Ipsos, 24 Feb. 2022). In order to convince the population to get vaccinated, the campaign “Pongo el hombro por el Perú” (I put my arm for Peru, in free translation) was launched, again appealing to patriotism. Likewise, the Ministry of Health promoted vaccination as a party and organized “vacunafests”, which included costume contests, dances, and concerts in the vaccination sites. By July-August 2021, the number of vaccinees increased dramatically, and covid-19 cases and deaths from it began to fall dramatically.

## Final considerations

The social responses in Peru to covid-19 were varied. We have shown how the population looked to religion, traditional remedies and charlatans for solutions to the health crisis caused by covid-19. At the same time, we have shown that a sector of the population feels distrust of science and health officials and thinks that behind these crises there are private or hidden objectives.

After the initial indifference, there were moments of panic (massive purchases of essential goods in supermarkets), search for culprits (there were some instances of anti-Asian racism, particularly criticism of the eating and hygiene habits in China) and blaming (those who went out to sell on the streets in the middle of the quarantine).

We can see a repetition of the past, in the fact that during the yellow fever epidemics in the nineteenth century and during the bubonic plague epidemics of the first decades of the twentieth century, the Chinese were also blamed for having brought these so-called “evils”.

Similarly, as in the case of the cholera epidemic in 1991, the less well-off sectors that had to go out to sell on the streets were blamed for the failure of social isolation measures. They were branded as dangerous populations, carriers and spreaders of the disease.

In the face of the strict quarantine decreed by the government in mid-March 2020 and extended until June of that year, the main response was resignation, even though it was a quarantine that involved drastic changes in the life of the population. As the months went by and it was not clear when the quarantine would end, the instances of resistance and disobedience became more notorious. Tens of thousands of people were seized or arrested in the country for throwing clandestine parties, playing soccer, or going out on the streets to trade informally during the quarantine. We need to remember that the strict quarantine lasted more than a hundred days, in which people could only leave their homes to go to the bank, to pharmacies or to markets. Schools, universities and offices were closed. Houses became homes, schools, and workplaces.

Finally, it should also be noted that, as has happened in previous pandemics, part of the population remained skeptic, stuck to the idea that the pandemic was nothing more than a smokescreen and an excuse for authoritarian measures to be taken and to sell vaccines. Likewise, as in previous cases, charlatans appeared with magical remedies and people resorted to prayer chains and the use of religious images as well. Despite significant scientific advances in recent centuries, a large sector of the population resorted to remedies and treatments not endorsed by health officials and scientists, such as ivermectin and chlorine dioxide. On the other hand, although there was some suspicion about Chinese vaccines because of the “vacunagate” scandal and the fake news, the vaccine was in demand and massively accepted.
